# COnCOmitaNt pulsed field ablation-based pUlmonary vein isolation and lefT atrial appendage closure: the COCONUT study

**DOI:** 10.1093/europace/euag198

**Published:** 2026-09-09

**Authors:** Christian-Hendrik Heeger, Laurent Roten, Domenico Della Rocca, Vedran Velagić, Dhiraj Gupta, Tom De Potter, Christian Sticherling, Lukas Urbanek, Boris Schmidt, Abdul Shokor Parwani, Ignacio Garcia-Bolao, Rocco Arancio, Amr Nawar, Shibu Mathew, Jennifer Beney, Henning Rolfes, Kolya Sievert, Serge Boveda, Antoine Lepillier, Marjorie Niro, Reza Wakili, Alexander Steger, Ahmad Awada, Vedran Pasara, Grégoire Massoullié, Andrija Nekic, Maarten Vanhaverbeke, Alexandre Almorad, Romain Eschalier, Maxim Didenko, Marco Contarini, Lorenz Räber, Jan-Per Wenzel, Manuel Rattka, Daniel Scherr, Roland Richard Tilz, Martin W Bergmann

**Affiliations:** Department of Cardiology, Asklepios Klinik Hamburg Altona, Germany, Paul Ehrlich Straße 1, 22763 Hamburg, Germany; Department of Rhythmology, University Heart Center Lübeck, University Hospital Schleswig-Holstein, Ratzeburger Allee 160, Lübeck, Germany; German Center for Cardiovascular Research, Partner Site Hamburg/Kiel/Luebeck, Potsdamer Str. 58, 10785 Berlin, Germany; Department of Cardiology, Inselspital, Bern University Hospital, University of Bern, Bern, Switzerland; Heart Rhythm Management Centre, Postgraduate Program in Cardiac Electrophysiology and Pacing, Universitair Ziekenhuis Brussel—Vrije Universiteit Brussel, Brussels, Belgium; European Reference Networks Guard-Heart, Laarbeeklaan 101, Brussels 1090, Brussels, Belgium; University Hospital Center Zagreb, Department of Cardiology, Zagbreb, Croatia; Liverpool Heart and Chest Hospital, Faculty of Health and Life Sciences, University of Liverpool, Liverpool, UK; Cardiovascular Center, AZORG Hospital Aalst, Aalst, Belgium; University Heart Center Basel, University Hospital Basel, Basel, Switzerland; CCB, Department of Cardiology, Frankfurt,Germany; Department of Cardiology, University Medical Center Mainz, Johannes Gutenberg-University Mainz, Mainz, Germany; CCB, Department of Cardiology, Frankfurt,Germany; Universitätsklinikum Frankfurt, Medizinische Klinik 3-Klinik für Kardiologie, Frankfurt, Germany; Charite, Deparment of Cardiology, Berlin, Germany; Clinica Universidad de Navarra, Department of Cardiology, Pamplona, Spain; Ospedale Umberto I, Department of Cardiology, Siracusa, Italy; Cairo University, Department of Cardiology, Egypt and SGH, Oman; Department of Cardiology, University Hospital Essen, Essen, Germany; Department of Cardiology, Inselspital, Bern University Hospital, University of Bern, Bern, Switzerland; Department of Cardiology, Asklepios Klinik Hamburg Altona, Germany, Paul Ehrlich Straße 1, 22763 Hamburg, Germany; CVC Frankfurt, Department of cardiology, Frankfurt, Germany; Clinic Pasteur, Department of Cardiology, Toullouse, France; Centre Cardiologique du Nord, Department of Cardiology, Saint Denis, France; Centre Cardiologique du Nord, Department of Cardiology, Saint Denis, France; Department of Cardiology/Vascular Medicine, University Heart and Vascular Center, Goethe University, Frankfurt, Germany; German Center for Cardiovascular Research DZHK, partner Site Rhine-Main, Germany; Department of Internal Medicine I, TUM School of Medicine and Health, Technical University of Munich, University Hospital, Munich, Germany; Department of Cardiology, CHU Brugmann, Vrije Universiteit Brussel, Brussels, Belgium; University Hospital Center Zagreb, Department of Cardiology, Zagbreb, Croatia; Cardiology Department, CHU Clermont-Ferrand, Clermont-Ferrand F-63000, France; Université Clermont Auvergne, CHU Clermont-Ferrand, CNRS, SIGMA Clermont, Institut Pascal, Clermont-Ferrand F-63000, France; University Hospital Center Zagreb, Department of Cardiology, Zagbreb, Croatia; AZ Delta, Department of Cardiology, Roeselare, Belgium; Heart Rhythm Management Centre, Postgraduate Program in Cardiac Electrophysiology and Pacing, Universitair Ziekenhuis Brussel—Vrije Universiteit Brussel, Brussels, Belgium; European Reference Networks Guard-Heart, Laarbeeklaan 101, Brussels 1090, Brussels, Belgium; Cardiology Department, CHU Clermont-Ferrand, Clermont-Ferrand F-63000, France; Université Clermont Auvergne, CHU Clermont-Ferrand, CNRS, SIGMA Clermont, Institut Pascal, Clermont-Ferrand F-63000, France; Department of Rhythmology, Herz- Gefäß- und Diabeteszentrum Bad Oeynhausen, Bad Oeynhausen, Germany; Ospedale Umberto I, Department of Cardiology, Siracusa, Italy; Department of Rhythmology, University Heart Center Lübeck, University Hospital Schleswig-Holstein, Ratzeburger Allee 160, Lübeck, Germany; Department of Rhythmology, University Heart Center Lübeck, University Hospital Schleswig-Holstein, Ratzeburger Allee 160, Lübeck, Germany; Department of Internal Medicine I, TUM School of Medicine and Health, Technical University of Munich, University Hospital, Munich, Germany; Division of Cardiology, Medical University of Graz, Graz, Austria; Department of Rhythmology, University Heart Center Lübeck, University Hospital Schleswig-Holstein, Ratzeburger Allee 160, Lübeck, Germany; German Center for Cardiovascular Research, Partner Site Hamburg/Kiel/Luebeck, Potsdamer Str. 58, 10785 Berlin, Germany; Department of Cardiology, Asklepios Klinik Hamburg Altona, Germany, Paul Ehrlich Straße 1, 22763 Hamburg, Germany

**Keywords:** Atrial fibrillation, Pulse field ablation, Left atrial appendage closure

## Abstract

**Aims:**

Atrial fibrillation (AF) ablation by pulmonary vein isolation (PVI) and left atrial appendage closure (LAAC) are increasingly performed as standalone procedures. Given that PVI and LAAC share the same access route, a combined approach of pulsed field ablation (PFA)-based PVI and LAAC might be beneficial. The COCONUT study focused on safety, efficacy, and outcomes combining PVI by PFA with LAAC in one procedure.

**Methods:**

This is a retrospective, multinational registry study. The primary endpoints were the following: (1) primary safety endpoint: serious adverse events; and (2) primary efficacy endpoint: success of PVI and LAA closure defined as the ability to isolate all pulmonary veins and the successful closure of the LAA. For secondary endpoints, peri-procedural data and follow-up data were collected.

**Results:**

A total of 155 patients from 22 centres of nine European countries were treated by concomitant pentaspline catheter PFA-based PVI and LAAC. The mean CHA^2^DS^2^-VA score was 3.1 ± 1.4. The primary efficacy endpoint was achieved at 97.4% with successful PVI at 100% and successful LAAC in 151/155 patients (97.4%). The primary safety endpoint was observed in 3/155 patients (1.9%). Transoesophageal echocardiography and/or CCTA 67 ± 26 days after the procedure showed no device embolization and no device-related thrombus, 24/151 (15.9%) patients showed peri-device gaps of <5 mm while gaps of >5 mm have been observed in 3/151 (2.0%). Among 137/155 patients with available arrhythmia, a follow-up >6 months at 78.1% showed sinus rhythm.

**Conclusion:**

In this large multicentre study, concomitant PFA-based AF ablation and LAA closure proved to be feasible, effective, and safe and overall procedure duration was short.

What’s new?

- This is one of the largest retrospective real-world cohorts evaluating a standardized concomitant workflow for PFA and LAAC performed during the same procedure.
- A single transseptal, low fluoroscopy strategy using pre-procedural CT planning enabled highly efficient combined therapy with excellent procedural success and low complication rates.
- Concomitant PFA + LAAC demonstrated high acute appendage seal rates, with low rates of clinically significant peri-device leak and rare device-related thrombus on follow-up imaging.
- Rhythm outcomes following integrated therapy showed favourable early rhythm observations, supporting the feasibility of an integrated ‘one-stop’ AF care model incorporating stroke prevention and rhythm control.

## Introduction

Atrial fibrillation (AF) ablation by pulmonary vein isolation (PVI) and left atrial appendage closure (LAAC) are increasingly performed as individual procedures. There are several studies which investigated a combined concomitant approach of performing a thermal-based PVI and an interventional LAAC in one procedure, and safety and efficacy have been shown.^1–4^ The recently published OPTION trial showed that, among patients who underwent thermal-based PVI, sequential or concomitant LAAC was associated with a lower risk of non-procedure-related major or clinically relevant non-major bleeding than oral anticoagulation (OAC) and was non-inferior to OAC with respect to a composite of death from any cause, stroke, or systemic embolism at 36 months.^5^ However, only thermal ablation has been utilized, and only 40.8% of patients have been treated concomitantly.^5^ The novel ablation modality pulsed field ablation (PFA) has significantly reduced procedure duration and potentially increased safety due to the specific ablation of cardiac tissue while sparing collateral tissue.^6^ With currently >500 000 treated patients, the most experience is currently available for the FARAPULSE PFA system (Boston Scientific). The MANIFEST-PF and MANIFEST-17K registries showed an excellent safety and efficacy profile for this novel ablation technology.^7,8^ In patients with non-valvular AF, at a high stroke risk, and who are ineligible for long-term OAC–LAAC could be an alternative to OAC.^9^ While PVI and LAAC are both conducted in the left atrium and share the same access route, a combined concomitant approach might be beneficial for patients but it is not conventionally practised.^10^ Due to significantly shorter procedure times and a high safety profile, PFA-based catheter ablation may be advantageous for a combined approach of PVI and LAAC. This might be beneficial, especially for patients with a high stroke and bleeding risk. The international multicentre COCONUT study was conducted to evaluate the safety, efficacy, and efficiency of combined PFA-based PVI and LAAC.

## Methods

### Study design

**Figure 1:**
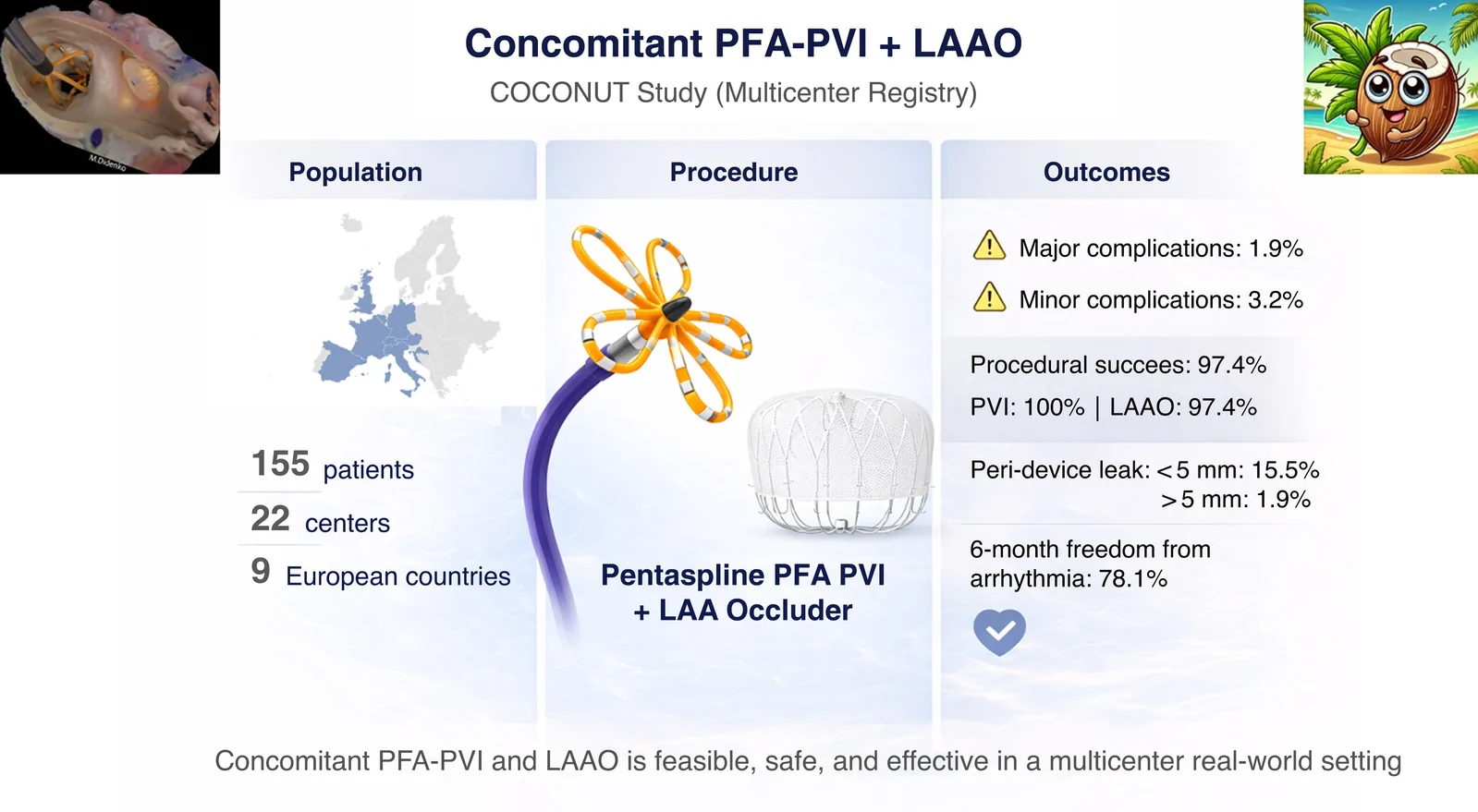
Summary of the COCONUT study results.

The COCONUT (COnCOmitaNt pulsed field ablation-based pUlmonary vein isolation and lefT atrial appendage closure) study is designed as a retrospective European-wide multinational, multicentre, anonymized registry study to evaluate the safety and efficacy of a concomitant PFA-based PVI and LAAC approach (registered at clinicialtrials.gov NCT06861673, *Figure 1*). The study was conducted at the Department of Cardiology at the Asklepios Klinik Altona in Hamburg, Germany. Experienced electrophysiological centres were invited to participate. The registry was approved by the local ethical review board of the Ärztekammer Hamburg, Germany (approval date 05.12.2024). Each participating centre was responsible for ethics approval by the local ethics committee. The study was performed in accordance with the ethical standards of the 1964 Declaration of Helsinki and its later amendments. All patient information was anonymized. The COCONUT study has been registered at clinicialtrials.gov (NCT06861673). Patients’ baseline characteristics, peri-procedural characteristics, and follow-up data were assessed according to a standardized and uniform questionnaire survey database. The acquired data was assessed for each individual patient. The inclusion criteria were patients with AF catheter ablation by PFA (Farapulse, Boston Scientific) and LAAC (WATCHMAN FLX or WATCHMAN FLX PRO, Boston Scientific). There were no exclusion criteria for the study.

### Procedure workflow

**Figure 2:**
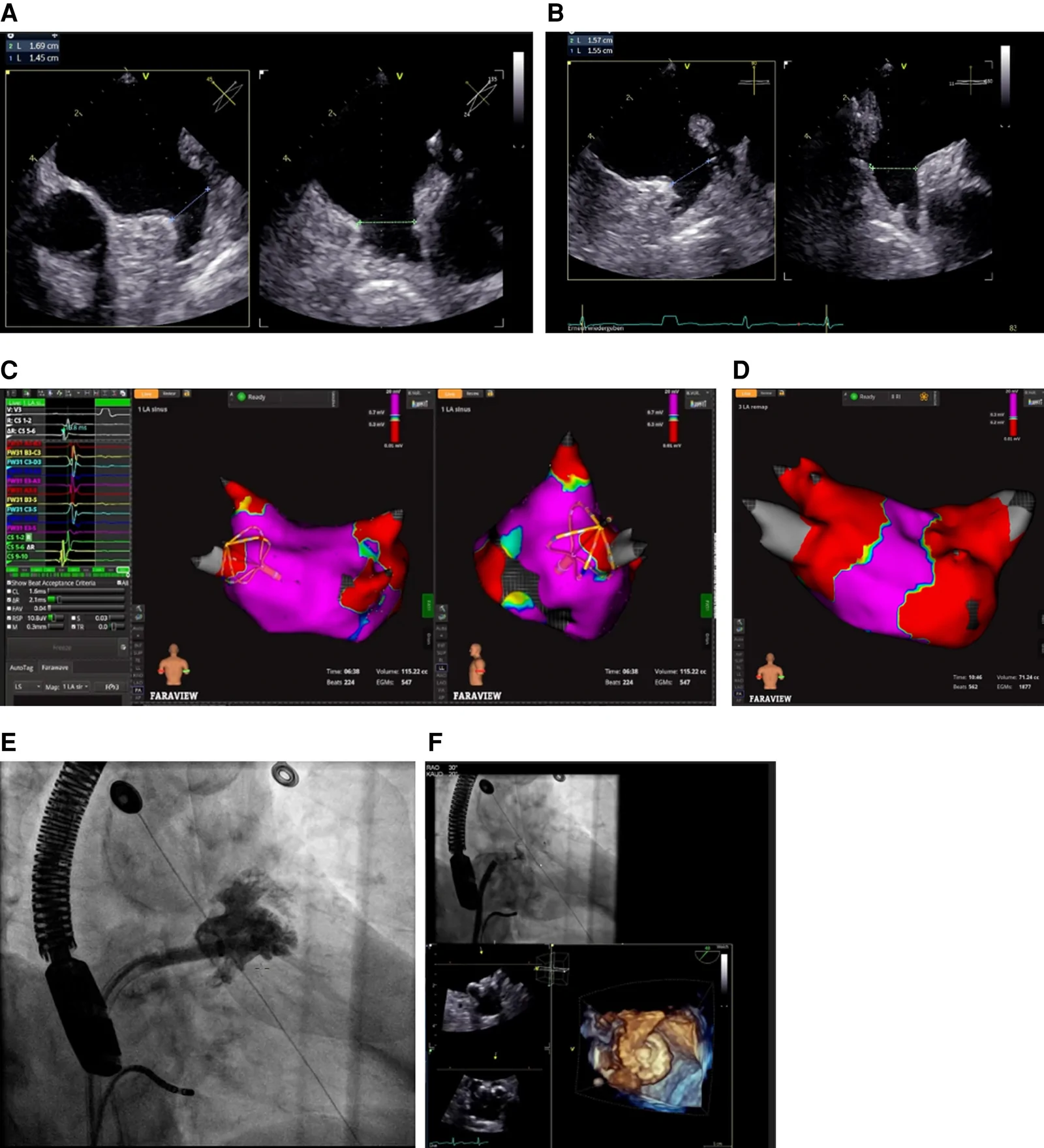
Workflow for concomitant PVI and LAAC. *A–B* Peri-procedural and pre-ablation imaging and measurements for LAAC. *C* Representative 3D electroanatomical mapping and PFA-based pulmonary vein isolation. The Farawave catheter is localized in the basket position at the ostium of the right inferior pulmonary vein. *D* Representative 3D electroanatomical map after pulmonary vein isolation with antral isolation of the pulmonary veins. *E* Angiography of the left atrial appendage via Watchman Sheath and *F* LAA closure via a 24 mm WATCHMAN FLX pro device.

All procedures were performed based on each individual centre’s protocols (*Figure 2*). A single transseptal puncture was used in all cases to facilitate PFA-based PVI and subsequent LAAC. Following transseptal access, the Faradrive sheath (16.8F outer diameter) was subsequently advanced through the same transseptal access for mapping and ablation. After the completion of PFA, a single sheath exchange was performed to a 15F outer diameter LAAC delivery sheath, and LAAC was performed according to each centre’s individual protocol. The post-procedural approach on OAC and antiplated therapy was based on each individual centre’s protocols.

### Study endpoints

The primary endpoints were the following: (1) Primary safety endpoint: major complications and serious adverse events (SAEs). The proportion of subjects with one or more of the following peri-procedural device- or procedure-related composite serious adverse events (CSAEs) assessed within 30 days following the index procedure, with an onset date following the procedure as specified: myocardial infarction, device embolism, severe vascular access complications, AV-block II or III, Gastric motility, pulmonary oedema, major procedural bleeding, death, stroke, TIA, cardiac tamponade or perforation, pericarditis, and systemic thromboembolism other than stroke.

(2) Primary efficacy endpoint: Success of PVI and LAA closure defined as the ability to isolate all pulmonary veins and successful closure of the LAA via a LAAC device. For secondary endpoints peri-procedural data, minor complications, and follow-up data were collected. All data acquisition is part of the clinical routine, and no extra examinations have been performed.

### Data management

Data were retrospectively and electronically collected. The analysis was performed using anonymized data only. The described data were retrospective data derived from the clinical routine of the participating centres, including routine follow-up visits. All members of the research team were obliged to secrecy. All data were protected from unauthorized external access, as only members of the research team were permitted and enabled to access these data.

### Statistical analysis

All categorical variables were reported as absolute, and relative frequencies were compared using Fisher’s exact test or the χ^2^ test. Continuous variables were tested for normal distribution using the Shapiro–Wilk test. They were reported as mean ± standard deviation (SD) in the case of normal distribution, otherwise as median and interquartile range (1st quartile, 3rd quartile). Continuous variables were compared using the non-paired Student’s *t*-test when normally distributed and the corresponding non-parametric test (Mann–Whitney *U* test) otherwise. All *P*-values are two-sided. Statistical significance was set at *P*-value < 0.05. All statistical analyses were performed using SPSS version 28.0 (IBM SPSS Statistics).

## Results

### Study population

A total of 155 patients (53.5% paroxysmal AF, mean age 69.5 ± 8.8 years) from 22 centres of 9 European countries were treated by concomitant PFA-based PVI and LAAC. The mean CHA^2^ DS^2^-VA Score was 3.1 ± 1.4, and the mean HASBLED score was 2.5 ± 1.0 (*Table 1*).

**Table 1:** Baseline patient characteristics

Variable	
Patients	155
Age, years	69.5 ± 8.8
Female gender	52 (33.5)
Paroxysmal AF	83 (53.5)
Congestive heart failure	26 (16.8)
Arterial hypertension	122 (79)
Diabetes mellitus	49 (31.6)
Coronary artery disease	55 (35.5)
Previous TIA/Stroke	33 (21.3)
Chronic kidney disease	37 (23.9)
Bleeding history	74 (47.7)
CHA_2_DS_2_-VA score	3.1 ± 1.4
0	0 (0)
1	12 (7.7)
2	46 (47.7)
3	41 (26.5)
≥4	56 (36.1)
HASBLED score	2.5 ± 1.0
0	0 (0)
1	29 (18.7)
2	39 (25.2)
3	65 (41.9)
≥4	22 (14.2)

Values are counts, *n* (%) or mean (±SD).

AF, atrial fibrillation; TIA, transient ischaemic attack

### Primary endpoints

The primary efficacy endpoint was 97.4%, with successful PVI of all pulmonary veins achieved in 100% (155/155) of patients after a mean of 34 ± 8 PFA applications and successful LAAC was achieved in 151/155 patients (97.4%). In detail, one patient showed massive LAA sludge after PVI, therefore, LAAC was successfully performed 4 weeks later after increased OAC and loss of the sludge. Three patients showed a complex two-lobe LAA anatomy that was deemed unsuitable for conventional LAAC employing the WATCHMAN FLX device.

The primary safety endpoint was observed in 3/155 patients (1.9%) with three major bleeding at the vascular access site. One patient experienced a severe arteriovenous fistula which was treated with surgical intervention. Two other patients experienced severe haematomas at the puncture site with prolonged hospital stays. No peri-procedural death, stroke, tamponade, and device embolization were observed.

### Secondary endpoints

All secondary endpoints are shown in *Table 2*. An ultrasound guided puncture was performed in 130/155 (83.9%) of cases. In 139/155 (89.7%) of cases, at least two sheaths have been used for the procedure, while in 12/155 (7.7%) patients, a single sheath approach has been utilized. Additional 3D mapping was performed in 72/155 (46.5%) procedures. All patients were treated with the FARAWAVE PFA Catheter (Boston Scientific) with 34/155 (21.9%) 35 mm size and 121/155 (78.1%) 31 mm size. Additional ablation beyond PVI was performed in 56/155 (36.1%) of cases mainly with posterior wall isolation (46/58, 79.3%). In 152/155 (98%), PVI was performed prior to LAAC. For LAAC-TEE, guiding was performed in 154/155 99% of cases while additional ICE guidance was reported in 19/155 (12.3%) cases. In 153/155 (98.7%) of cases, the LAA landing zone measurements were conducted before PVI.

**Table 2:** Procedural details

Variable	
Number of patients	155
Total procedure time, min	79.7 ± 30.1
Total fluoroscopy time, min	17.4 ± 8.7
DAP cGy/cm^2^	1464 ± 1946
PVI-first approach	152 (98.1)
LAA closure first	3 (1.9)
LAA sizing prior PVI	153 (98.7)
*Catheter ablation*	
Number of PVs	620
Total number of isolated PVs	620 (100)
PFA only	620 (100)
31 Farawave PFA catheter	121 (78.1)
35 mm Farawave PFA catheter	34 (21.9)
Farawave Nav PFA catheter	10 (6.5)
3D mapping guided approach	72 (46.5)
Mean number of PFA application/pts.	34 ± 8
Additional posterior wall isolation	46 (29.7)
Additional LAA isolation	6 (3.9)
Additional CTI ablation	6 (3.9)
Additional slow pathway ablation	1 (0.6)
*LAA closure*	
Acute LAA sealing	151 (97.4)
Device size change	9 (6.0)
Device embolization	0 (0)
Device leakage <5 mm	4 (2.6)
Device leakage >5 mm	0 (0)
Device dislocation	0 (0)
WATCHMAN FLX	141 (93.4)
WATCHMAN FLX pro	10 (6.6)
LAA closure device distribution	
20 mm	24 (15.9)
24 mm	48 (31.8)
27 mm	52 (34.4)
31 mm	21 (13.9)
35 mm	6 (4.0)
40 mm	0 (0)
*Post-procedural oral anticoagulation*	
NOAC	139 (92)
Warfarin	3 (2)
Low molecular weight heparin	2 (1.3)
Single antiplateled therapy	4 (2.6)
Dual antiplateled therapy	3 (2)

Values are counts, *n* (%) or mean (±SD).

CTI, cavotricuspid isthmus; DAP, dose area product; LAA, left atrial appendage; PFA, pulsed-field ablation; PV, pulmonary vein; PVI, pulmonary vein isolation

While mainly the WATCHMAN FLX was utilized, 10 patients received the latest-generation WATCHMAN FLX Pro (Boston Scientific) LAAC device with modified fabric coating (10/151, 6.6%). The mean procedural duration was 80 ± 30 min. At the end of the procedure, residual device leaks (<5 mm) were observed in 4/151 (2.6%) patients while no leaks of >5 mm was observed. A complete sealing was achieved in 151/155 patients (97.4%).

The minor complication rate was 3.2% (4/155); in all four cases, a minor bleeding at the puncture site was reported (*Table 3*).

**Table 3:** Safety outcomes

Variable	
Severe adverse events	3 (1.9)
Cardiac tamponade	0 (0)
Severe bleeding at puncture site	3 (1.9)
Phrenic nerve injury	0 (0)
Stroke or TIA	0 (0)
Esophageal injury	0 (0)
Acute kidney failure	0 (0)
Minor complications	5 (3.2)
Minor bleeding at puncture site	5 (3.2)
Pericardial effusion	0 (0)

Values are presented as *n* (%). Major bleeding was defined as bleeding associated with haemodynamic compromise, transfusion, procedural intervention, a decrease in haemoglobin ≥2 g/dL, bleeding at a critical site, or prolonged hospitalization.

TIA, transient ischaemic attack

No additional peri-procedural difficulties have been reported by the operators.

### Post-procedural findings and follow-up

Antiplatelet therapy or OAC was prescribed according to each centre’s protocols. Transoesophageal echocardiography and/or CCTA 67 ± 26 days after the procedure showed no device embolization or dislocation (*Table 4*).

**Table 4:** Follow-up imaging and rhythm outcomes

Variable	
Time to follow-up imaging, days	67 ± 26
No leakage	130 (86.1)
Leakage <5 mm	24 (15.5)
Leakage >5 mm	3 (1.9)
Device related thrombus	0 (0)
Device dislocation	0 (0)
Available arrhythmia follow-up >6 months	137 (88.3)
Stable Sinus Rhythm at follow-up >6 months	107 (78.1%)
Stroke during follow-up	0 (0)
Bleeding during follow-up	2 (1.5)

Values are presented as *n* (%).

At follow-up, 24/151 (15.9%) patients showed peri-device gaps of <5 mm while gaps of >5 mm were observed in 3/151 (2.0%) of cases. Among 137/155 patients with available arrhythmia follow-up of > 6 months (mean follow-up 185 days), 78.1% showed sinus rhythm.

During 6-month the follow-up no strokes were reported. Two patients (2/137 1.5%) experienced gastro-intestinal (GI) bleeding during follow-up.

## Discussion

The COCONUT study is the first multicentre study exploring the safety and efficacy of combined PFA and LAAC. The main findings of the COCONUT study are: (1) A one-stop strategy of concomitant PFA-based PVI and LAAC can be safely and effectively performed in a single session within a relatively short time. (2) Most operators chose a PVI-first approach, and performing PVI prior to LAAC may minimize the risk of catheter–device interaction. (3) Measurement of LAA dimensions prior to PVI seems to be essential to avoid device undersizing in the presence of post-ablation tissue oedema.

The OPTION trial showed that interventional LAAC in patients after PVI (either sequential or concomitant) was non-inferior to OAC (composite of death from any cause, stroke, or systemic embolism at 36 months) and was associated with a lower risk of non-procedure-related major or clinically relevant non-major bleeding in comparison to OAC.^5^ From the recent perspective, there are several limitations: Only thermal-based PVI has been utilized and only 40.8% of patients have been treated concomitantly. With a massively reduced procedure duration and increased safety, PFA has significantly impacted catheter ablation procedures. A combined concomitant strategy of PFA-based PVI and LAAC might be a suitable option for patients to treat AF and prevent stroke and bleeding with one single procedure. Today, only case reports and small registries are available for concomitant PFA-based PVI and LAAC.^10–12^ Currently, there are several unanswered questions including safety, effectivity, feasibility, patient selection, device selection, order of the procedure, and reimbursement considerations.

### Safety and efficacy of a concomitant strategy

The primary efficacy endpoint of successful PVI and LAAC was achieved in 98% of patients and is comparable with the latest findings.^5^ This reflects the technical feasibility of a combined approach and is in line with the findings of sequential and concomitant strategies.^5,13^ A complete sealing of the LAA was achieved in 97.4% of patients, which is similar to the results of a recent sub-analysis of the OPTION study. For both concomitant and sequential ablation timing strategies, a complete sealing was similar and have been achieved in 97.3% (concomitant) and 97.1% (sequential), respectively.^13^ At follow-up, the rate of complete sealing was reduced to 86.1%, with leakages of <5 mm in 15.5% and leakages of >5 mm in 1.9% of cases. Although the number of leakages increased at follow-up, the findings are comparable to findings of the OPTION study where complete sealing was detected in 78.6–80.3%. Yet, further advances in imaging and possibly newer device generations might abandon leaks following LAAC in a combined approach.

With 1.9% of procedures, a low rate of major complications was observed, showing a high safety for the combined strategy which was even lower in comparison to the OPTION study (2.8%).^5^ Minor complications were reported in 3.3%. It is remarkable to state that all major and minor complications are vascular access site-related and no additional typical complications of either LAAC or catheter ablation occurred. Most likely, this is reflecting the good safety profile of PFA and potentially the high rate of US-guided puncture (83.9%) observed in this study. With the limitation to just one single procedure, the concomitant approach seems to be equally effective compared to a sequential approach and beneficial in terms of safety.

### Order of the concomitant approach

The order of a concomitant PVI-LAAC strategy is currently controversial. On one hand, ablation on a previously implanted LAAC device could potentially impair the device;^14^ on the other hand, an undersized LAAC device due to potential ablation-induced oedema at the ridge area might be an issue. Although concomitant thermal ablation-based PVI and LAAC showed feasibility and safety, some studies and long-term observations reported a higher incidence of peri-device leaks on follow-up in patients undergoing concomitant thermal ablation followed by LAAC.^15,16^ This phenomenon is most likely related to post-ablation tissue oedema at the ridge area, which may result in the underestimation of LAA dimensions and subsequent LAAC device undersizing. Supporting this hypothesis, an occlusion-first approach was previously suggested.^17^ With non-thermal PFA, tissue oedema might not affect the LAAC procedure. Some studies have reported on the mild swelling of the ridge after PFA-based PVI.^18,19^ However, very limited peri-procedural difficulties were reported by the operators of the COCONUT study, a PVI-first approach mainly was performed (98%) and the LAA dimension measurements have been mainly conducted prior to PVI (98.7%). With different PFA and LAAC devices, it is debatable whether there are differences in oedema formation and impairment of the LAAC device during ablation. This appears to be particularly relevant with the two-component LAAC devices, which are located at the ridge area with the potential risk of contact to the PFA catheter during energy delivery when ablating at the left pulmonary veins.^12^ In the COCONUT study, only the Farapulse, the WATCHMAN FLX, and WATCHMAN FLX pro LAAC devices have been studied. Performing mainly a PVI-first strategy and conducting the landing zone measurements mainly prior to PVI, no post-procedural peri-device leakages, dislodgement, device-related thrombus, or impairment of the LAAC device were found. To prevent the impairment of the LAAC via PFA energy, we suggest a PVI-first strategy with pre-procedural imaging and sizing of the LAA dimensions e.g. via CT and peri-procedural confirmation of the measurements via TEE or ICE to allow for optimal LAAC results regarding position and sealing.^20,21^

### Patients’ perspective of a concomitant approach

The advantages of a single combined procedure in patients with an indication for both treatments are the potential reduction of peri-procedural complications, higher convenience for the patients, but also economic and reimbursement factors also play a role. Recent findings suggest the termination of OAC after successful PVI in patients with limited stroke risk based on the CHA_2_DS_2_-VA score might be possible.^22,23^ However, it is important to state that these studies only included patients with a relatively low risk and are observing a short follow-up period. The fact that the PVI is proven to massively reduce the AF-burden also needs to be taken into account when performing a procedure with aimed at the stroke risk. However, PVI is not able to cure AF yet. Furthermore, the lifetime risk for stroke based on the CHA_2_DS_2_ -VA score is not a stable situation, but rather a dynamic and individual increase over time. Therefore, the treatment of AF in combination with preventing stroke and bleeding by a single one-stop procedure seems to be a valuable long-term option for patients with AF, risk for stroke, and bleeding.^24^ A recent OPTION sub-analysis of concomitant and sequential ablation timing strategies found that LAAC has similar efficacy compared with OAC and a lower risk of clinically important post-procedure bleeding following AF ablation.^13^ Especially for patients with a high bleeding risk and contraindications for OAC, the OAC therapy could be terminated about ∼2 months after successful PVI and LAAC, and bleeding episodes could potentially be avoided. From patients and economic perspective, the limitation of one hospital stay has several advantages in comparison to two hospital stays. However, in some countries there are reimbursement considerations when performing a concomitant procedure.

### Concomitant approach for strategies beyond PVI

Besides the observed positive effects, a concomitant approach might also include to electrically isolate the LAA prior to LAAC. Several studies identified the LAA as a potential target for catheter ablation via LAA isolation especially in patients not responding to PVI.^25,26^ Due to the high risk for thrombus formation and stroke after LAA isolation, LAA occlusion via interventional LAAC is crucial.^27,28^ A staged sequential approach might be a potential risk for patients with LAA isolation and a concomitant strategy might be beneficial. Recent findings on long-term durability after PFA-based LAA isolation showed very poor results in comparison to radiofrequency and cryoballoon-based techniques.^29^ A recent case report showed that PFA is able to treat LAA-originated atrial tachycardias (AT) and durably isolate the LAA, followed by successful LAAC. Until today, only very limited data are available for LAA isolation and LAAC and this might be a future target for catheter ablation and LAAC in patients not responding to PVI.

### Staged vs. concomitant approaches

A staged approach, in which PVI (with subsequent reassessment of the LAA after the resolution of a potential post-ablation oedema), may improve device sizing accuracy and reduce the risk of undersizing or peri-device leak. The principal disadvantage of this strategy is the requirement for continued OAC during the interval between procedures. By contrast, performing LAAC before ablation may subject the implanted device to potential electrical or mechanical interactions during subsequent ablation and may render transseptal re-access more technically challenging. Each sequencing strategy therefore presents distinct advantages and limitations; selection should be individualized according to patient characteristics and bleeding risk. Prospective studies are warranted to determine the optimal procedural sequence and the ideal timing of imaging in combined pulsed-field ablation and left atrial appendage closure.

## Limitations

The COCONUT study has several limitations. First, the findings were based on a retrospective analysis. Nevertheless, the data was obtained from a large number of centres across Europe and represents the largest database on a concomitant PVI plus LAAC up to date. Second, not all data were available and some patients were lost to follow-up. Third, a comparison to a propensity score matched cohort would be beneficial and could potentially show better insights on this novel treatment strategy. Finally, our findings are only reflecting the data based on the combination of the Farapulse and WATCHMAN FLX devices. Although both systems are the devices with the most clinical utilization, there are several other systems available with potentially different advantages and disadvantages for concomitant procedures.

## Conclusion

A streamlined concomitant PFA-based PVI and LAAC procedure utilizing the Farapulse and WATCHMAN FLX devices seems to be feasible and safe. Even as PFA swelling of the ridge has been previously observed, the sizing measurements have been performed prior to PVI and the LAAC procedure was successful within the long-term follow-up with a minimal number of patients with relevant LAA leakage. A concomitant approach might be a suitable option for selected patients.

## Data Availability

The data underlying this article will be shared on reasonable request to the corresponding author.
